# Differences in the distribution of risk factors for stroke among the high‐risk population in urban and rural areas of Eastern China

**DOI:** 10.1002/brb3.461

**Published:** 2016-04-07

**Authors:** Te Mi, Shangwen Sun, Yifeng Du, Shougang Guo, Lin Cong, Mingfeng Cao, Qinjian Sun, Yi Sun, Chuanqiang Qu

**Affiliations:** ^1^ Neurology DepartmentShandong Provincial Hospital Affiliated to Shandong UniversityJinanShandong250021China; ^2^ Cardio‐Cerebrovascular Control and Research CenterInstitute of Basic MedicineShandong Academy of Medical SciencesJinanShandong250062China; ^3^ Medical DepartmentShandong Provincial Hospital affiliated to Shandong UniversityJinanShandong250021China

**Keywords:** Distributional differences, Eastern China, high‐risk population, risk factors, stroke, urban and rural areas

## Abstract

**Introduction:**

Considering the program of screening for risk factors of stroke in Eastern China, the aim of this study was to compare the distribution differences in risk factors for stroke among the high‐risk population living in urban and rural areas.

**Methods:**

A total of 231,289 residents were screened and basic information collected. Risk factors for stroke among the high‐risk population were compared between the urban and rural groups.

**Results:**

A total of 117,776 high‐risk residents from urban areas and 113,513 from rural areas were included in the analysis. The prevalence of hypertension was much higher in rural areas (73.3%) than that in urban areas (64.1%). Dyslipidemia (48.9% vs. 26.9%), sport lack (46.6% vs. 31.6%), diabetes mellitus (21.3% vs. 16.5%), and atrial fibrillation (18.7% vs. 9.8%) were more prevalent in the urban group, while smoking (26.5% vs. 28.8%), previous stroke (10.1% vs. 16.9%), and transient ischemic attack (20.9% vs. 24.6%) were less prevalent.

**Conclusion:**

Among the population at high risk of stroke, there were significant differences in the distribution of the following risk factors between the urban and rural groups: hypertension, atrial fibrillation, dyslipidemia, lack of physical exercise, and a previous stroke.

## Introduction

Stroke is a leading cause of acquired disability worldwide (The World Health Organization 2013) and is the second highest global cause of mortality (Mathers et al. 2009). Recurrence of stroke is associated with an increased risk of death, further disability, and dependence on health services (Prencipe et al. 1998). In China, stroke has become the major cause of death (Sun et al. 2013a; Feigin et al. 2003). The *China Health Statistical Yearbooks* (Wang 2000) revealed that the distribution of stroke mortality was different between urban and rural areas. Since 2006, stroke mortality has been higher in rural areas compared with urban areas. In China, the urban population is more likely to have a higher education level, a higher socioeconomic status, and are able to use healthcare according to their needs (Chau 2010; Shi et al. 2011). Similarly, in a study of 100,000 people living in high‐, middle‐, and low‐income countries, Yusuf et al. (2014) observed that case fatality and major cardiovascular events were more common in rural areas, but the risk factor burden was greater in urban regions. Interestingly, in high‐income countries, event rates were similar in urban and rural regions, probably owing to similar access to the health system in these regions. Therefore, higher stroke mortality in rural areas of China may be the result of reduced access to rapid diagnostics, inappropriate drug treatment, and poorer educational levels.

As the Chinese lifestyle has changed rapidly, with economic and societal growth during the past three decades, the prevalence of risk factors (RFs) for stroke in China has approached those of Western countries (Li et al. 2015). Hypertension, diabetes mellitus, hypercholesterolemia, smoking, atrial fibrillation (AF), physical inactivity, obesity, and a family history of stroke are the major factors for the risk assessment of stroke (based on the National Health and Family Planning Commission of Stroke Screening and Prevention Projects). Usually, there are multiple RFs (more than three) in high‐risk stroke populations. Recent studies have shown that RFs including obesity and hypercholesterolemia have substantially increased in both urban and rural areas (Critchley et al. 2004; Pang et al. 2005); especially the mean blood cholesterol level (Pang et al. 2005). The incidence of chronic diseases such as hypertension, diabetes mellitus, and AF differs considerably between urban and rural areas (Zhai et al. 2009; Liu et al. 2006). Furthermore, the prevalence of hypertension has decreased in urban male individuals, but increased in rural male individuals. In rural female individuals, the prevalence of all vascular RFs except smoking has decreased. According to the National Health and Family Planning Commission of Stroke Screening and Prevention Projects, a risk assessment for stroke and stroke screening of urban and rural residents was implemented in Eastern China from 2011 to 2012. As RFs included in the assessment are reported to be associated with the prognosis of stroke survivors (Liu et al. 2006; Mathisen et al. 2016), various treatments should be taken in view of the recognition of different RFs.

The aim of this study was to assess differences in the distribution of RFs for stroke between the high‐risk population living in urban and rural areas in Eastern China and to provide further recommendations for stroke management and prevention for the high‐risk population.

## Materials and Methods

### Ethics statement

The study was conducted according to the guidelines of the Helsinki Declaration. Ethical approval was obtained prior to the start of the study from the Ethics Committee of Shandong Provincial Hospital affiliated to Shandong University. Written informed consent was obtained from all participants.

### Study population

The study population and the proportion of the population to screen were determined according to the following criteria. First, provinces were selected to ensure the convenient transportation and the feasibility to make a long‐time follow‐up of high‐risk groups. Second, the proportion of the target population for screening in each region was determined according to the sixth census urban and rural resident population (age >40 years). Proportional screening was undertaken according to the age distribution and sex ratio of the sixth census of the population in each province. According to the standard of permanent resident population referred in the sixth census statistics, urban residents include the population in a city divided into districts and the town population in a city not divided into districts, and the rest of the population is rural residents. Lastly, cluster sampling was used in 18 urban regions and 18 rural regions of Shandong Province (with a large population and rapid economic development), the representative province of Eastern China.

The number of individuals screened within each region was no fewer than 6000. The 231,289 permanent residents over 40 years old (date of birth between 1 January 1937 and 31 December 1971) in screening locations were eligible for screening. Residents living for ≥6 months in the area were also included in our study, but recent migrants between urban and rural areas were not considered for inclusion. All participants provided informed consent.

### Data collection

**Figure 1:**
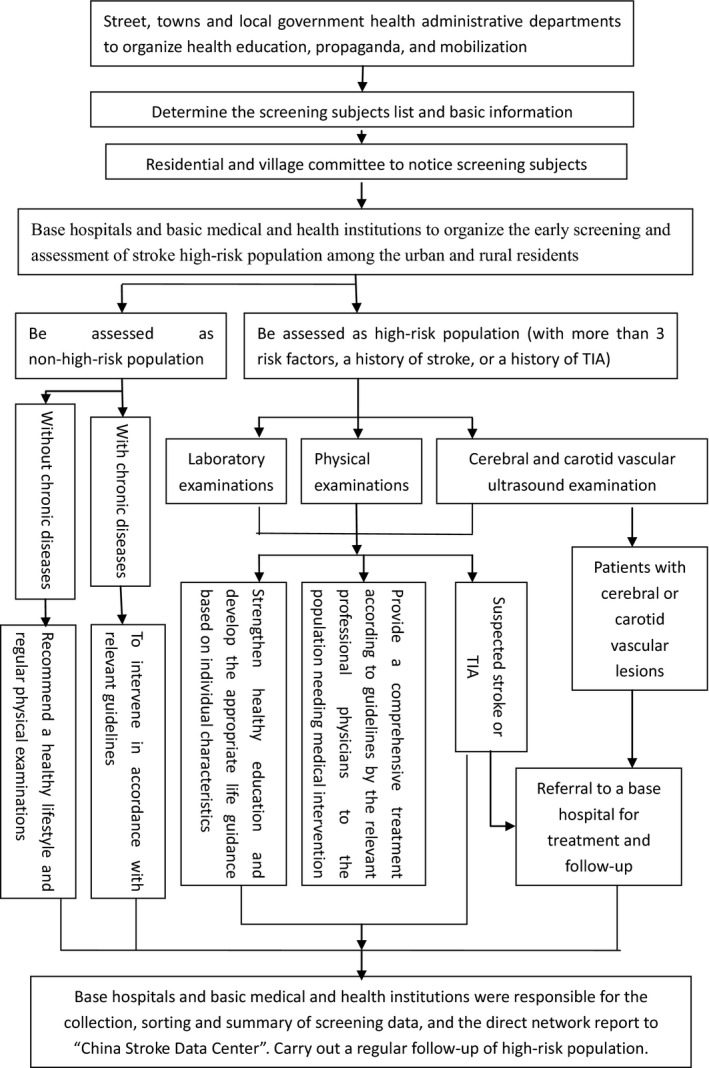
Flow chart showing study inclusion criteria and work‐ups.

All data were collected using a nationally agreed questionnaire. Figure 1 showed the work steps.

### RFs for stroke risk assessment

According to the screening and intervention workflow for the population at high risk of stroke, early screening was implemented based on an assessment of ten RFs.

### Category I RFs

RF1: hypertension, defined as a history of high blood pressure (≥140/90 mmHg) reported by the participant or the current use of antihypertensives. RF2: AF reported by the participant or indicated by electrocardiogram. RF3: diabetes mellitus defined by previous diagnosis, treatment with insulin/oral hypoglycemic medications, or a fasting plasma glucose level ≥126 mg/dL or glycosylated hemoglobin ≥6.5%. RF4: dyslipidemia defined as the current use of anti‐lipidemic medication, total cholesterol ≥5.7 mmol/L, serum triglyceride ≥1.7 mmol/L, or low‐density lipoprotein ≥3.1 mmol/L. RF5: smoking defined by either the current or former practice of smoking. RF6: lack of physical exercise defined as physical exercise <3 times a week and each less than 30 min in duration (industrial and agricultural labor was considered as physical exercise). RF7: overweight, defined as a body mass index ≥25 kg/m^2^. RF8: self‐reported family history of stroke.

### Category II RFs

RF9: a previous history of stroke determined by a previous diagnosis. RF10: history of transient ischemic attack (TIA) determined by a previous diagnosis.

### Risk stratification

According to the preliminary screening table of stroke risk formulated by the National Health and Family Planning Commission of Stroke Screening and Prevention Project, high‐, medium‐, and low‐risk populations were defined as follows. High‐risk individuals were defined as participants with three or more category I RFs, one category II RF, or one or more RFs in category I, and category II. Participants with fewer than three category I RFs and chronic diseases (hypertension, diabetes mellitus, or AF) were classified as medium risk. Participants having three or fewer category I RFs and no chronic diseases were classified as low risk.

### Statistical analysis

Descriptive characteristics of study subjects according to their urban and rural area of residence were reported as percentages for categorical variables and mean ± SD for continuous variables. The Student *t*‐test was performed to assess differences in age. The chi‐square test was used to compare frequencies of sex, education level, and stroke risk assessment between the urban and rural residents. Binary logistic regression modeling was used to estimate the association of hypertension, diabetes mellitus, dyslipidemia, AF, smoking, and other RFs with living in urban areas for high‐risk groups after adjustment for age and sex. The results were expressed as multivariable‐adjusted odds ratios (ORs) and 95% confidence intervals (95% CIs). A two‐sided *P *<* *0.05 was considered statistically significant. All data analyses were performed, using SPSS 18.0 (Jinan, China) statistical analysis software.

## Results

Descriptive characteristics of the study population are shown in Table 1. A total of 231,289 residents in Shandong Province of Eastern China were included in the analysis. The 117,776 residents in the urban area had a mean (SD) age of 56.7 (11.5) and 54,777 (46.5%) were men. Between the urban and the rural populations there was no significant difference in mean age (*P *=* *0.171), but there were significant differences in sex distribution (*P *=* *0.005), education level (*P *<* *0.05), and stroke risk assessment (*P* < 0.05). The urban population was at a higher risk level for stroke, with a lower proportion of men, and a higher level of education.

**Table 1:** Descriptive characteristics of the urban and rural populations

Category	Urban	Rural	*P*
	(*n* = 117,776)	(*n* = 113,513)	
Age (mean ± SD)	56.7 ± 11.5	56.7 ± 11.2	0.171
Male (*n*, %)	54,777 (46.5)	53,454 (46.8)	0.005
Education level (*n*, %)			<0.05
≤Primary school	47,550 (40.4)	81,314 (71.6)	
Middle school	37,305 (31.7)	28,778 (25.4)	
High school	21,873 (18.6)	222 (0.20)	
Stroke risk assessment (*n*, %)			<0.05
Non‐risk	67,608 (57.4)	67,749 (59.7)	
Low‐risk	13,836 (11.7)	12,565 (11.1)	
Medium‐risk	16,668 (14.2)	14,898 (13.1)	
High‐risk	19,664 (16.7)	18,301 (16.1)	

The distributional differences in stroke RFs between the urban and the rural high‐risk groups were specifically analyzed (Tables 2 and 3). As shown in Table 2, hypertension was the most common risk factor for stroke, but the prevalence of hypertension was much higher in rural areas (73.3%) than that in urban areas (64.1%). There were significant differences in the distribution of all RFs except overweight (*P *=* *0.822) and family history of stroke (*P *=* *0.783). Dyslipidemia, lack of physical exercise, diabetes mellitus, and AF were more prevalent in the urban group (48.9%, 46.6%, 21.3%, and 18.7%, respectively) than those in the rural group (26.9%, 31.6%, 16.5%, and 9.8%, respectively). In contrast, smoking, previous stroke, and TIA were less prevalent in the urban group (25.6%, 10.1%, and 20.9%, respectively) than those in the rural group (28.8%, 16.9%, and 24.6%, respectively). After multivariable analysis (Table 3), the distributional differences were statistically significant for the following stroke RFs: hypertension (OR: 0.690, 95% CI: 0.651–0.732), AF (OR: 1.913, 95% CI: 1.779–2.057), dyslipidemia (OR: 1.341, 95% CI: 1.309–1.374), lack of physical exercise (OR: 1.523, 95% CI: 1.444–1.607), and previous stroke (OR: 0.533, 95% CI: 0.497–0.573). Variables included in the logistic regression model (AF, dyslipidemia, lack of physical exercise) were more prevalent among the urban residents.

**Table 2:** Risk factors for stroke in high‐risk populations

	Urban	Rural	
Risk factors	(*n *= 19,664)	(*n* = 18,301)	*P*
Hypertension (*n*, %)	12,595(64.1)	13,411(73.3)	<0.05
AF (*n*, %)	3,670(18.7)	1,788(9.8)	<0.05
Diabetes (*n*, %)	4,196(21.3)	3,017(16.5)	<0.05
Dyslipidemia (*n*, %)	9,611(48.9)	4,918(26.9)	<0.05
Smoking (*n*, %)	5,038(25.6)	5,263(28.8)	<0.05
Sport lack (*n*, %)	9,168(46.6)	5,778(31.6)	<0.05
Overweight (*n*, %)	10,072(51.2)	9,396(51.3)	0.822
Family history of stroke (*n*, %)	5,735(29.2)	5,313(29.0)	0.783
Previous stroke (*n*, %)	1,992(10.1)	3,097(16.9)	<0.05
Previous TIA (*n*, %)	4,105(20.9)	4,496(24.6)	<0.05

**Table 3:** Associations between risk factors for stroke in high‐risk populations^a^

Risk factors	Odds ratios (OR)	95% CI
	(urban vs rural)	
Hypertension	0.690	0.651–0.732
AF (atrial fibrillation)	1.913	1.779–2.057
Diabetes	1.186	1.114–1.264
Dyslipidemia	1.341	1.309–1.374
Smoking	0.894	0.834–0.958
Sport lack	1.523	1.444–1.607
Previous stroke	0.533	0.497–0.573
Previous transient ischemic attack	0.853	0.802–0.907

a Binary logistic regression analysis adjusted for age and sex. The rural group was the reference.

## Discussion

After adjustment for multiple co‐factors, we found significant differences in the distribution of a number of RFs for stroke (hypertension, AF, dyslipidemia, lack of physical exercise, and previous stroke) between urban and rural high‐risk groups.

Our study found that the mean age of the high‐risk population was 56.7 years; suggesting that the onset age of stroke appeared to be lower than the normal health. A study of Chinese stroke patients found that the prevalence of stroke was significantly different by sex and region and more than half of first incidences of stroke occurred before the age of 60 years (Zhai et al. 2009). With changes in dietary and lifestyle habits among the Chinese population, people are at a higher risk of stroke and other vascular events. It is therefore necessary to carry out early education on the RFs for cardiovascular disease and apply appropriate preventative measures among the Chinese population.

Although stroke mortality and case fatality have been declining in Eastern China, RFs such as hypertension, diabetes mellitus, obesity, and smoking have become more prevalent and are poorly controlled (Kim 2014). In this study, hypertension and being overweight were found to be the most common RFs in the high‐risk population, consistent with previous studies (Liu 2007, 2011; Yuan et al. 1998; Ezzati et al. 2003). Our study also showed that the prevalence of hypertension in urban areas was much lower than that in rural areas. The pronounced difference in the distribution of hypertension (OR: 0.690, 95% CI: 0.651–0.732) between urban and rural areas might be owing to the community control of hypertension in the 20th century (Liu 1989); sodium restriction (Chen et al. 2008); higher rates of treatment, and improved awareness of hypertension (Hu et al. 2000) in urban areas. Although no distributional difference was found in the prevalence of overweight between the two groups, this risk factor has become more prevalent in both urban and rural China as a result of changes in lifestyle and diet (Li et al. 2015). For example, the prevalence of being overweight or obese increased by 85% in rural areas and 13% in urban areas between 1994 and 2002 (Zhao et al. 2008).

Studies published in the 1990s suggested that the incidence of stroke in rural China was low (Xue et al. 1991; He et al. 1995). However, studies have shown that the mortality rate of stroke has gradually decreased since the 1990s, more so in urban areas than that in rural areas for the middle‐aged and older populations. In this study, previous stroke (OR: 0.533, 95% CI: 0.497–0.573), as the major complication of hypertension, was also found at a higher prevalence among the rural high‐risk group. Similarly, the prevalence of TIA (OR: 0.853, 95% CI: 0.802–0.907) was higher in rural areas than in urban areas. As rural China has been undergoing rapid social and economic change during the past two decades, especially in eastern China, rural residents periodically commute to urban regions where they have acquired Western lifestyle and dietary habits (Du et al. 2001; Sun et al. 2013b). The incidence of hospitalized AF was significantly lower in non‐white populations including the Chinese, and a large proportion of AF appears to be attributable to hypertension among non‐white populations (Rodriguez et al. 2015). In our study, after adjusting for age and sex, the prevalence of AF (OR: 1.913, 95% CI: 1.779–2.057) was found to be significantly higher in the urban group than that in the rural group. The previously reported RFs for AF in Western cohorts (including coronary heart disease, obesity, and alcohol consumption – Kodama et al. 2011) can also be found in the Chinese population (Li et al. 2013a). The higher prevalence of AF in urban areas might be caused by differences in working, environmental, and lifestyle factors between urban and rural areas. However, there are not enough published studies to verify whether the associated RFs have caused the different distribution of AF between the urban and rural groups.

A lack of physical exercise (OR: 1.523, 95% CI: 1.444–1.607), dyslipidemia (OR: 1.341, 95% CI: 1.309–1.374), and diabetes mellitus (OR: 1.186, 95% CI: 1.114–1.264) was found to be more common in the urban high‐risk population, while distributional differences for the first two RFs were more apparent. Changes in occupations, the advent of new technologies, and the rapid pace of urban life have increasingly resulted in more sedentary work and less energy expenditure. Unlike Western people, urban Chinese have low participation in exercise/sports (Shi et al. 2013). Rural Chinese also had little participation in leisure time activities, but most are manual workers. As a result, working practices of the rural population are equivalent to physical exercise, while the urban population, most of whom did not have physically intensive jobs, participated in less sport.

In addition to the results of this study, the higher prevalence of dyslipidemia has also been found in a number of recent studies in Chinese urban areas (Zhao et al. 2007; Wang et al. 2012; Mai et al. 2014). Dyslipidemia has become one of most important RFs threatening the health of Chinese people, with hypertriglyceridemia and low blood high‐density lipoprotein‐cholesterol as the two major types (Zhao et al. 2005). A combination of lipid abnormalities, hypertriglyceridemia, and low high‐density lipoprotein is metabolically interlinked and has been collectively termed “atherogenic dyslipidemia” (Grundy and Vega 1992; Vega 2004). Increasing dyslipidemia is primarily driven by nutrition, lifestyle, and demographic transitions, increasingly faulty diets, and physical inactivity (Misra and Shrivastava 2012). People with a lower socioeconomic status may also have a lower total food (calorie) intake, especially of animal proteins and are more likely to consume vegetables (Hu et al. 2002). For example, during the past three decades, total cholesterol intake increased from 124.8 to 350.7 mg/day in rural areas and from 334.5 to 488.4 mg/day in urban areas (Pang et al. 2005). Fortunately, some studies (Arboix et al. 2010; Biffi et al. 2011) have demonstrated that the use of statins before a first‐ever ischemic stroke is associated with a better early outcome and reduced mortality. It is necessary to promote the application of statins in the population at high risk of stroke.

As for smoking, a distributional difference in this risk factor (OR: 1.523, 95% CI: 1.444–1.607) was also found in this study. Overall awareness of the health hazards of tobacco has improved in the previous 15 years in China, but is still relatively poor, particularly among rural residents and people with lower levels of education (Yang et al. 2010), which might explain the higher proportion of smoking in the rural areas.

Considering all RFs together, the probable mechanisms leading to the different distributions of these RFs for stroke between the urban and rural high‐risk populations can be explained as follows. First, urban Chinese are more likely exposed to a number of challenges such as imbalanced diets, physical inactivity, long working hours, and other urban stress, making them vulnerable to chronic diseases, including dyslipidemia, diabetes mellitus, and cardiovascular diseases (Mai et al. 2014). Second, there is a strong association between lower socioeconomic position and risk for hospitalization for stroke (Andersen et al. 2014), and most of the rural Chinese population has both a lower income and lower education levels. Furthermore, a lack of public knowledge of stroke (Sun et al. 2011), poor management of vascular RFs (Zeng et al. 2012; Li et al. 2013b), and a reduced awareness of stroke guidelines in community physicians (Niu et al. 2014) combine to contribute to the current situation.

The strengths of the study are the large representative sample of both urban and rural populations in eastern China, a full consideration of important stroke RFs, and an analysis of possible reasons resulting in the different distributions of RFs.

However, when interpreting these findings, several limitations of this study should be considered. First, there was a certain limitation in age. Only people aged ≥40 but <80 years were included. Second, there is a possible selection bias for the screening population owing to the influence of geographic location, society, and the form of the questionnaire survey. Third, binary logistic regression modeling was implemented to analyze RFs independently, without considering interactions between them. Finally, we are unable to conduct further in‐depth analyses of certain RFs, such as the particular form of sport, the abnormal types of dyslipidemia, and total times of previous stroke.

Despite the aforementioned limitations, this study still significantly contributes to the understanding of stroke high‐risk populations in Eastern China by exploring some of the differences in the prevalence of stroke RFs among urban and rural groups. Our results indicate significant differences exist in most RFs across the two high‐risk populations, with greater AF, dyslipidemia, and lack of physical exercise in the urban group and more hypertension and previous stroke in the rural population. These findings suggest that health promotion and education of the risks of stroke should be enhanced in Eastern China, especially for high‐risk populations. Further understanding of these differences will help design tailored education and RF management programs in specific communities with the ultimate goal of stroke prevention.

## Conflict of Interest

None declared.
